# Spider traps amphibian in northeastern Madagascar

**DOI:** 10.1002/ece3.7102

**Published:** 2020-12-11

**Authors:** Thio Rosin Fulgence, Dominic Andreas Martin, Holger Kreft, Fanomezana Mihaja Ratsoavina, Aristide Andrianarimisa

**Affiliations:** ^1^ Natural and Environmental Sciences Regional University Centre of the SAVA Region (CURSA) Antalaha Madagascar; ^2^ Zoology and Animal Biodiversity, Faculty of Sciences University of Antananarivo Antananarivo Madagascar; ^3^ Biodiversity, Macroecology and Biogeography University of Goettingen Goettingen Germany; ^4^ Centre for Biodiversity and Sustainable Land Use (CBL) University of Goettingen Goettingen Germany

**Keywords:** amphibians, behavior, *Heterixalus andrakata*, Madagascar, predation, spider

## Abstract

Predation can take unexpected turns. For instance, various invertebrate species—most commonly spiders—may prey on vertebrates. Here, we report one observation of a spider (Sparassidae, *Damastes* sp.) feeding on an amphibian (Hyperoliidae, *Heterixalus andrakata*) inside a retreat in northeastern Madagascar. To our knowledge, this is the second report of vertebrate predation by spiders in Madagascar. Three additional observations of retreats built by the same spider species show that the spiders built similar retreats and were hiding at the rear end of the retreat. The retreats were built by weaving two green leaves together which were still attached to the tree. We speculate from the observations, that the retreat serves as a targeted trap that deceives frogs seeking shelter during daytime.

## Graphical abstract

We report a observation of a spider (Sparassidae, *Damastes* sp.) preying on an amphibian (Hyperoliidae, *Heterixalus andrakata*) at a retreat built by the spider in northeastern Madagascar. One case of the report is the spider feeding on a small frog, and three additional reports are concerning spiders hide at the rear end of retreat. We speculate from the observations, that the retreat serves as a targeted trap that deceives frogs seeking shelter during daytime.

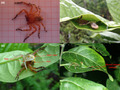

## 1 INTRODUCTION

Finding food is an important component of animal behavior, encompassing on average more than 50% of a lifetime activity budget (Fennessy, 2004). Predation is an important technique to acquire food (Bertram, 1979; Kie, 1999) and occurs between many different taxa, such as vertebrates preying on other vertebrates, for example, a bird preying on a gecko (Koski & Merçon, 2015; Lopes et al., 2005), snakes feeding on lizards (Raselimanana, 2018), and amphibians eating amphibians (Glaw & Vences, 2007; Ndriantsoa et al., 2014; Rasolonjatovo et al., 2018), or between vertebrates and invertebrates, for example, a bird eating a butterfly (Bowers et al., 1985; Collins & Watson, 1983; Olofsson et al., 2010; Pinheiro & Cintra, 2017; Stefanescu, 2000; Su et al., 2015). However, invertebrates can also prey on vertebrates, thereby turning the “expected order” around. Reported cases are geographically widespread and highly diverse: for example, crabs preying on frogs (Pyke et al., 2013; Rosa et al., 2014), dragonfly larvae (Barej et al., 2009) and water scorpions eating tadpoles (von May et al., 2019), water bugs preying on fish (von May et al., 2019), praying mantis feeding on lizards (Jehle et al., 1996), and carabid beetles feeding on amphibians (Wizen & Gasith, 2011). These case and further examples are collated in a recently published review (Valdez, 2020).

The review also reveals that spiders are among those invertebrate predators which have been reported to prey on vertebrates and different types of prey by spider have been listed in several papers such Jackson (1987); Pekár et al. (2012); Michalko and Pekár (2016). Among those vertebrate, preying by spider are mammals (von May et al., 2019), reptiles (Folt & Lapinski, 2017; von May et al., 2019; Shine & Tamayo, 2016), and amphibians (Amaral et al., 2015; Costa‐pereira et al., 2010; Folly et al., 2017; Gaiarsa et al., 2012; Glos, 2003; Kirchmeyer et al., 2017; von May et al., 2019; Menin et al., 2005; Pedrozo et al., 2017). Generally, amphibians seem to be the most common vertebrate prey of spiders (Valdez, 2020), probably due to their soft skin (Valdez, 2020), but also due to their small to moderate size (Duellman & Trueb, 1986).

Most reports have documented spiders to catch their prey by active hunting (Kirchmeyer et al., 2017; Maffei et al., 2010) or by using orb webs to catch flying or jumping vertebrates such as bats, birds, and amphibians in midair (Folt & Lapinski, 2017; Kirchmeyer et al., 2017; Muscat et al., 2014; Nyffeler & Kno, 2013; Toledo, 2005).

Here, we report on a predation by a spider of the genus *Damastes* sp. catching a small frog (*Heterixalus andrakata,* Glaw and Vences, 1991, Least Concern) in northeastern Madagascar. As we understand, this is the second report of spider predation on amphibians in Madagascar.

## 2 MATERIALS AND METHODS

### 2.1 Study area

**Figure 1:**
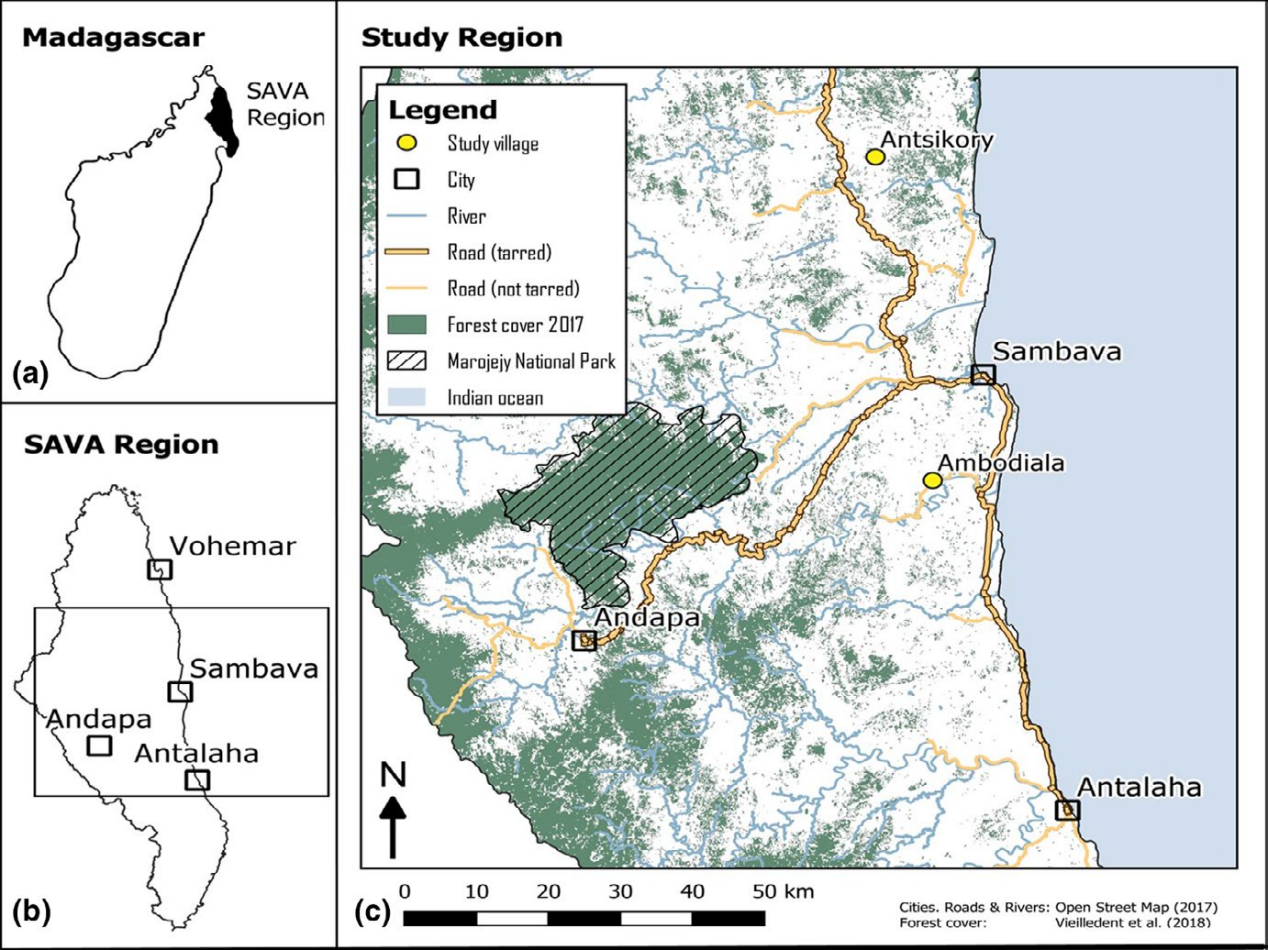
Study area. (a) Location of the SAVA region in northeastern Madagascar, (b) General study region where the observations were conducted and (c) Location of Ambodiala and Antsikory, the villages where the observations were conducted

We conducted field observations around Ambodiala (commune Farahalana, Sambava District) and Antsikory (commune Ampanefena, Vohemar District) in northeastern Madagascar (Figure 1). The climate in this part of Madagascar is tropical‐humid. The landscape was formerly covered with humid evergreen forests (Du Puy & Moat, 1996), but forests are nowadays fragmented (Vieilledent et al., 2018) and the landscape is dominated by smallholder agriculture.

### 2.2 Incidental observations

We made four incidental observations during ecological surveys in the study area. DAM made the first incidental observation, at which the spider was feeding on the amphibian, in the morning on 25th October 2017 in a woody fallow in Ambodiala (14°24′47″ S, 50°5′17″ E) after a bird point count. The woody fallow is a former slash‐and‐burn *(tavy)* field on which rice was last cultivated in 2001. The shrubs and trees inside the woody fallow were around two to three meters high.

All other observations, at which only the retreat was observed, were made by TRF inside vanilla plantations during additional ecological surveys in the area. The second observation was on 20th August 2018 at 7:40 p.m. in Antsikory (13°55′35.8″ S, 50°02′40.1″E). The third observation was on the same date at 9:00 p.m. in the same village but in a different vanilla plantation (13°55′49.0″ S, 50°02′26.3″E). The fourth observation was on 3rd October 2018 at 6:34 p.m. in Ambodiala (14°24′28″ S, 50°5′8″E). Vanilla plantations in the study region represent agroforestry systems characterized by vanilla vines growing on small‐statured support trees, while tall trees provide shade.

### 2.3 Specimens

**Figure 2:**
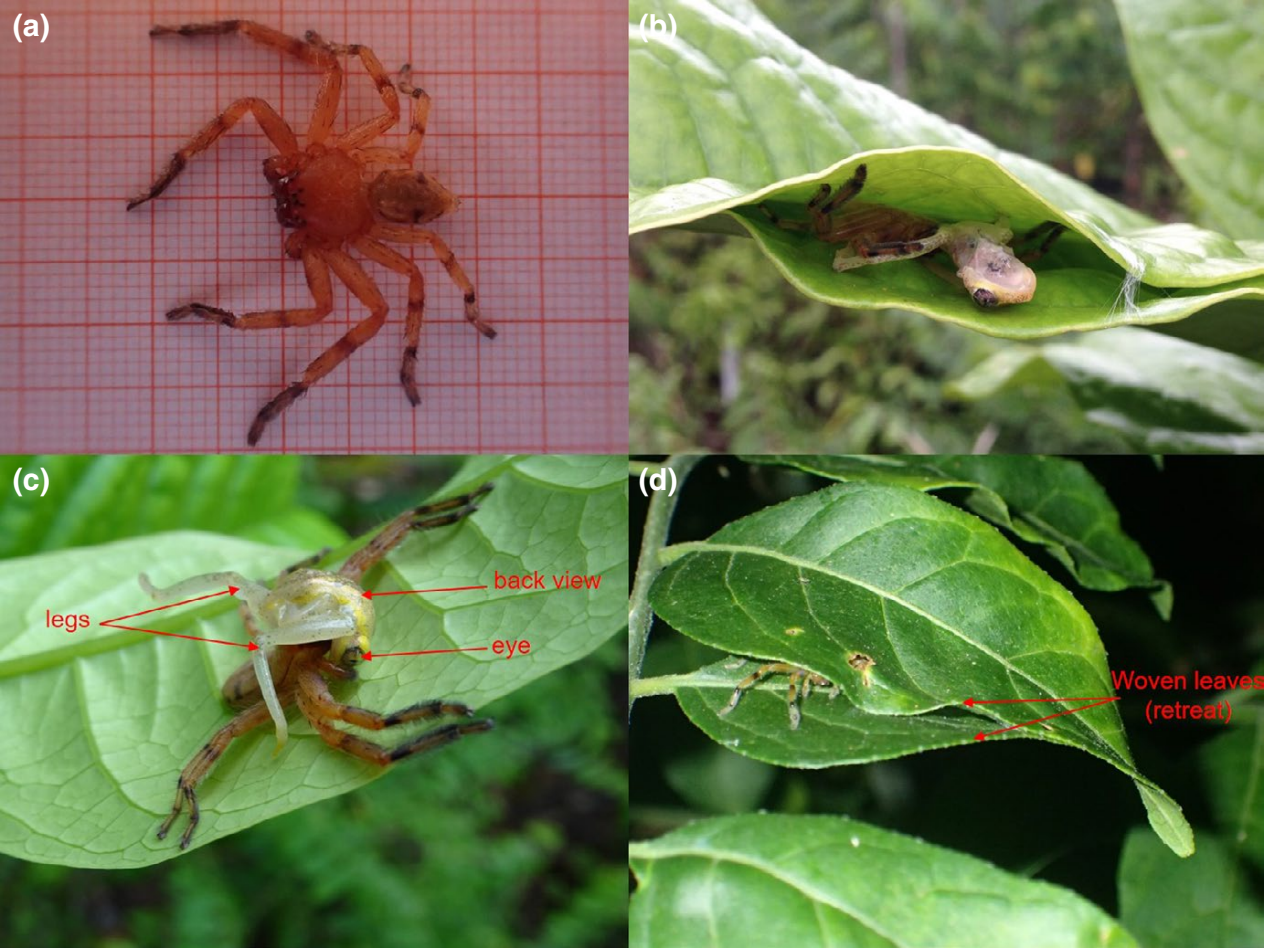
Retreat and predation event near retreat of *Damastes* sp. (a) Spider specimen of *Damastes* sp. (THC140, adult female), the prosoma and opisthosoma are approximately 1.5 cm in length (smallest square = 0.1 cm)—Observation 1; (b) *Damastes* sp. feeding on *Heterixalus andrakata* (frog) inside of the retreat, built of leaves of *Tambourissa* sp.—Observation 1, (c) Predation event where *Damastes* sp. captured *Heterixalus andrakata* near the retreat—Observation 1; (d) *Damastes* sp. hiding in the retreat, built of leaves of *Cedrela odorata*—Observation 4

Two spider individuals were collected, euthanized, and fixed in 90% ethanol. We labeled voucher specimens with field numbers THC140 (first observation) and THC293 (fourth observation). We measured the specimen THC140 on millimeter paper (Figure 2a) to record prosoma and opisthosoma length. While we have not collected the frog specimen observed during the predation, we have collected one individual from the same locality of the same species, which was recorded with the field number THC144. It has been euthanized, fixed with 90% alcohol, conserved in 70% alcohol, and stored at the University Center of SAVA Region (CURSA). Tissue biopsies of frogs and spider specimens, preserved in 90% alcohol, were also deposited at the Evolutionary Biology laboratory at the University of Braunschweig, Germany. We verified the frog identification based on DNA sequences of the 16S rRNA gene of the Mitochondrial DNA. We identified the spiders to genus level with the help of an expert in arachnology, Dr. Peter Jäger, from the Senckenberg Research Institute and the Natural History Museum in Frankfurt, Germany.

## 3 RESULTS

We found four different spider retreats from *Damastes* sp. (Sparassidae) that were built using leaves in three different species of trees. In all cases, the retreats consisted of leaves woven and pulled together with a spider silk. Thereby, the leaves became close to each other, closing roughly two thirds of the leaf edges. The leaves were still green and attached to the stem. The leaves were woven on apex and edge (Figure 2d), only on edges (Figure 2b) and in middle of the segmented of each other (case of *Phyllarthron madagascariensis*). The retreats were open at the leave's base and the spiders were well‐hidden at the rear end of the trap (i.e., the apex of the leaves) and not visible from the entrance.

### 3.1 First incidental observation

After a bird point count in the morning (6:45 a.m.), we saw how a spider (*Damastes* sp.) was feeding on a frog (*Heterixalus andrakata*, Hyperoliidae) near the woven leaves of *Tambourissa* sp. The spider held on the head of the amphibian with the fangs. The amphibian posterior legs were above the back of the spider, while the head was down. The amphibian did not move anymore, so it seemed already killed (Figure 2c). When we approached the scene, the spider with prey went hiding into the retreat (Figure 2b). We took photographs and left the predation event. The tree leaves measured circa 26 cm in length and circa 9 cm in width at the widest point. The height of the leaves from the ground was around 120 cm.

In the afternoon (4:15 p.m. of the same day), we came back to the same place and the spider was still at the same place (hiding between the leaves). We collected the specimen (Figure 2a) but could not find the frog prey anymore. Around the tree, within a 2‐m‐radius, we found four other living individuals of *Heterixalus andrakata*. During the second through fourth incidental observations, we found the same spider species hiding between leaves of different tree species but we could not observe any predation events.

### 3.2 Second incidental observation

We found the spider during a nocturnal amphibian and reptile survey in a vanilla plantation hiding in the retreat built in two leaves of *Phyllarthron madagascariensis*. The tree leaves measured circa 29 cm in length and circa 8 cm in maximum width. The height of the woven leaves where the spider was hiding was around 180 cm from the ground.

### 3.3 Third incidental observation

The third observation resembled the second, but occurred in a different vanilla plantation within the same village, circa 300 m away from the second observation. The height of the woven leaves of *Phyllarthron madagascariensis* where the spider was hiding was around 170 cm above the ground.

### 3.4 Fourth incidental observation

We found the spider hiding between leaves of *Cedrela odorata* (Figure 2d). Before we took the picture, we found the spider at the far end of the retreat. When we took the picture, the spider was flushed out from the retreat. The length of the leaf was circa 8 cm with a width of circa 3 cm. We found the woven leaves around 50 cm from the ground.

## 4 DISCUSSION

### 4.1 Predation event and retreat

We report one predation event of spider *Damastes* sp. eating a small frog *Heterixalus andrakata*. Additionally, we observed three individuals of the same species of spider sitting in retreats made by green leaves attached to the stem of the tree. The retreats generally show the same shape. Two leaves were woven by spider silk in the apex, edges and open in the base of the leaves seems enabling prey climbing up the stem of the tree to enter. The spiders do not seem to have a preference for a single tree species and the height from the ground also seems variable from those observations.

Spiders are the most cited invertebrate group preying on vertebrates (Barej et al., 2009). However, the majority of reports of amphibian predation by invertebrates stems from the Neotropics. Few predation events on Afrotropical anurans by invertebrates have been published (Babangenge et al., 2019). Reports from Africa are from Tanzania and Uganda, where fishing spiders prey on tadpoles (Vonesh, 2005), from South Africa, where crabs predate on amphibians, and from Cameroon, where wandering spiders prey on tree frogs (Barej et al., 2009). Whether this geographic bias concerning amphibian predation by invertebrates is indeed reflecting a difference in the frequency of such behavior or whether the bias is due to more research being conducted in the Neotropics (Martin et al., 2012012; Meyer et al., 2015) remains, however, unclear. Our observation is, to our knowledge, the second report of spider predation on vertebrates from Madagascar after Glos (2003), which reports on spiders (Pisauridae) feeding on frogs (*Heterixalus tricolor*) on the reed grass stems within a pond in Kirindy dry forest.

### 4.2 Characteristics of *Damastes* sp. spiders and *Heterixalus* sp. frogs

The genus *Damastes* is included in the Sparassidae family, a group that is called “huntsman spiders” (Rayor, 2018) and occurs around the world (Jäger, 2012). Most huntsman spiders do not build webs to capture their prey (Rayor, 2018). Instead, some of them are known for their running and hunting habit. But, the genus *Damastes* is an exception since members of the genus typically use a sit‐and‐wait approach (Soutinho et al., 2018). However, some Sparassidae have been found in their own silk nest which is fastened with debris, leaving leaves or stems that are completely surrounded by silk (Jackson, 1987). Furthermore, most species in the family Sparassidae are nocturnal (Rayor, 2018).

The genus *Heterixalus* is predominantly arboreal and typically occurs in open areas within the human‐dominated landscape (Blommers‐Schlösser, 1982; Raharivololoniaina et al., 2003). The species *H. andrakata* is distributed in northern and northeastern Madagascar (Glaw & Vences, 2007). During our ecological survey, we found *H. andrakata* to be mostly active at night, but recorded some daytime activity in agroforests. However, the species is typically hiding away during daytime between leaves, possibly to avoid dehydration.

### 4.3 Speculation on possible systematic trapping behavior

Previous reports of spiders preying on amphibians point to an opportunistic behavior and provide no evidence of specialization. Based on our report, we speculate that the spiders use targeted traps to prey on amphibians. We base this speculation on several strings of evidence. First, some reports describe spider retreats used by spiders as a protection from predators (Henschel & Jocqué, 1994; Nentwig & Heimer, 1987; Stradling, 1994; Thirunavukarasu et al., 1996). However, these retreats may also be modified to serve as a trap (Nentwig & Heimer, 1987). Second, the behavior was observed independently in four spider individuals at four different sites suggesting that the retreat building is frequently performed by *Damastes* sp. in northeastern Madagascar. Third, a key factor facilitating the trapping behavior of *Damastes* sp. may be that *Heterixalus andrakata* and possibly also other arboreal frogs try to hide from sunlight during the day in order to avoid dehydration (Rodel & Braun, 1999). When temperatures rise, the frogs look for shade and cover away from the ground, which the spiders provide in form of their retreat. The frogs might favor the seemingly protected traps in an attempt to hide from other predators such as birds that scan the vegetation for prey. Based on these strings of evidence, we speculate that amphibians may not only be an opportunistic, indiscriminate, or accidental prey, but rather a targeted systematically exploited food source of *Damastes* sp. spiders.

Nonetheless, further research is required to confirm this, especially so, as we only report a single observation of the spider feeding on the frog. Additionally, large prey such as the frog is more likely to catch the attention of an observer, thereby posing the risk of being overinterpreted.

## CONFLICT OF INTEREST

The corresponding author declares on behalf of authors that there is no conflict of interest to disclose.

## AUTHOR CONTRIBUTIONS

**Thio Rosin Fulgence:** Conceptualization (equal); methodology (equal); validation (equal); writing‐original draft (lead). **Dominic Andreas Martin:** Conceptualization (equal); methodology (equal); validation (equal); writing‐original draft (equal). **Holger Kreft:** Conceptualization (equal); methodology (equal); supervision (equal); validation (equal); writing‐original draft (supporting). **Fanomezana Mihaja Ratsoavina:** Conceptualization (equal); methodology (equal); supervision (equal); validation (equal); writing‐original draft (supporting). **Aristide Andrianarimisa:** Conceptualization (equal); methodology (equal); supervision (lead); validation (equal); writing‐original draft (supporting).

## Data Availability

Data sharing is not applicable to this article as no new data were created or analyzed in this study.
